# Exploring the possible sources of fiscal space for health in India: insights from political regimes

**DOI:** 10.1186/s12961-022-00831-4

**Published:** 2022-03-24

**Authors:** Deepak Kumar Behera, Umakant Dash, Santosh Kumar Sahu

**Affiliations:** 1 Department of Commerce, Manipal Academy of Higher Education, Manipal, 576104 India; 2 Institute of Rural Management, Gujarat, 388001 India; 3 Department of Humanities and Social Sciences, Indian Institute of Technology Madras, Chennai, 600036 India

**Keywords:** Fiscal space, Health financing, Health policy, Political regimes, Political commitment, India

## Abstract

**Background:**

Rising healthcare costs and poor access to health services have become a significant concern for policy-makers; therefore, efforts must be made to generate fiscal space through alternative revenue measures in resource-poor economies. This study attempts to identify possible sources of fiscal space for health in India across political regimes.

**Methods:**

The study followed a descriptive approach to examine the political commitment towards health sector development by estimating the trend of growth in fiscal space indicators over the political regimes from 1998–1999 to 2021–2022 using a dummy variable regression model.

**Results:**

We found four possible sources of fiscal space for health, which include (1) raising domestic revenue mobilization, (2) generating alternative revenue collection mechanisms, (3) prioritizing health through expenditure management and (4) effective utilization of central transfer. Fiscal space measures such as goods and services tax reform, collection of health-specific tax, higher excise duty on tobacco products, cooking gas subsidies to poor people, tax administration reform and direct beneficiary transfer of health services could be alternative revenue mobilization channels for fiscal space for health.

**Conclusion:**

The study reveals that the central government has a political commitment to generating revenue through various fiscal policy reforms. Health has been prioritized over the period, but there is less evidence of health-related political commitment for an increased share of health expenditure to total budgetary allocation. During the last 2 years, however, the health budget has been prioritized due to the COVID-19 pandemic crisis despite slower economic growth in India. This study will be a policy document for fiscal space analysis from a political-economic perspective, and the role of the ministry of finance can be assessed through administrative data and documents.

## Background

Fiscal space refers to the capacity of the government to provide additional budgetary resources for the desired purpose without jeopardizing the sustainability of its long-term financial position [1]. Generating fiscal space for the health system, thereby achieving universal health coverage, is the foremost objective of the United Nations Sustainable Development Goals [2]. Many committees have been set up since 2000 to strengthen the health system across the globe, including the Commission on Macroeconomics and Health in 2001, the Taskforce on Innovative International Financing for Health Systems in 2009 the Health Systems Financing Strategy Report of 2010, and the Chatham House report on Shared Responsibilities for Health of 2015 [3]. These committees have concluded that sustainable health financing policies through the generation of fiscal space in resource-poor economies are required for the overall development in the health sector.

Past studies argue that resource-poor economies, including India, often struggle with lower fiscal space for health, leading to a poor health system [4, 5]. In the case of India, many studies have suggested various fiscal policy channels for revenue mobilization for health, including compulsory funding through taxation, the contribution from the organized sector (through income tax) and specific central transfers [6, 7]. In this context, a few have argued that innovations in resource mobilization may have little impact without the strengthening of public healthcare systems, and innovation in information and communications technology (ICT) can improve access to health services [8]. Despite all these efforts, India is considered a lower-health-spending country because around 70% of the population pay their medical bills as out-of-pocket expenditure, which is lower than other Asian countries [9]. India has not even achieved the 12th Five-Year Plan target to increase public health spending to 2–3% of gross domestic product (GDP) by 2015, which has shown less political will over the last two decades [10].

Against the above backdrop, this study explores the possible sources of fiscal space for government health expenditure and the performance of fiscal space for health parameters in India. The fiscal space analysis for health is highly relevant due to the following factors. First, the provision of the health budget allocation is the primary responsibility of the state government. Still, most Indian states mainly depend on central government resources, including central tax share and central grants to states. Second, there is less evidence on whether conducive macroeconomic conditions lead to better resource mobilization to states. Existing literature argues for a high reduction in central government funding for the Indian case [6, 9]. Third, the central government has been trying to reduce the fiscal deficit to around 3.5% of GDP as per the Fiscal Responsibility and Budget Management Act (FRBM), reducing social services sector spending [11]. Fourth, as per the Fifteenth Finance Commission recommendation, the central tax share to states is increased from 32 to 42%. Still, the states’ prioritization of health expenditure is less than that for other expenses [12].

Based on the above arguments, we examine the political commitment towards the health sector by estimating the growth trend in fiscal space indicators over the political regimes from 1998–1999 to 2020–2021. We adopted four political regimes at the central government level—the National Democratic Alliance 1 (NDA1, 1998–1999 to 2003–2004), United Progressive Alliance 1 (UPA1, 2004–2005 to 2008–2009), UPA2 (2009–2010 to 2013–2014) and NDA2 (2014–2015 to 2020–2021). The NDA is a centre-right coalition of political parties in India. The UPA is a coalition of centre-left political parties in India formed after the 2004 general election. We adopted revenue mobilization indicators that include tax revenue, nontax revenue, central grants, central tax share to states, and borrowings as per the intertemporal budget constraint criteria for health [4]. Additionally, we adopted expenditure prioritization of health vis-à-vis other sectoral expenditures. We used data from various secondary sources that include the Indian Public Finance Statistics reports and the annual budget report of the Ministry of Finance [13, 14]. We also synthesized government documents that include budget speeches of the Ministry of Finance and an economic survey of the Government of India [13–15] to identify the health sector development and financial commitment of government regimes in power over the past two decades.

The remainder of the paper is organized as follows. The related literature section discusses the analytical framework of fiscal space for health and country-level experiences in generating fiscal space for health using existing literature. The methodology section provides the empirical methods and the descriptive results. The results section discusses the empirical results. Finally, the last section discusses fiscal policy measures and commitment to health sector development in India and concludes with possible policy suggestions.

## Related literature

This section presents the analytical framework of fiscal space for health and generating fiscal space for health at the country level. We divide this section into two parts. The first part explains the theoretical understanding of fiscal space for health, and the second part presents country-level experiences in the generation of fiscal space for health.

### Theoretical understanding of fiscal space for health

The concept of fiscal space for health generation has gained a significant place in policy discussions in the international forum. It is the only channel through which a country can generate finance for healthcare and probably move forward to achieve universal health coverage [16, 17]. The fiscal space for health argument was initially popularized by Heller [1], Tandon and Cashin [4], and Durairaj and Evans [5]. They identified five channels through which resource-poor economies could generate fiscal space for health: tax revenue mobilization, prioritization of expenditure on health, health-specific taxes, health sector-specific grants/foreign aid, and efficiency of government expenditure. The first three channels are identified as general macro-fiscal policies, and the last two channels fall within the domain of the health sector.

We use intertemporal budget constraint criteria to explain the sources of fiscal space for health as suggested by Tandon and Cashin [4, 18]. We present the intertemporal budget identity in Eq. (2), where we explain that fiscal space can be generated through four sources: taxation (*T*_*t*_), borrowings (*B*_*t*_), grants (*A*_*t*_) and other sources of revenue (*O*_*t*_). The right-hand side of Eq. (2) represents the total budgetary revenue, and the left-hand side represents the total budgetary expenditure. Budgetary expenditure includes government noninterest expenditure (*G*_*t*_) and nondiscretionary debt interest payments (*B*_*t*−1_). Further, the fiscal space for health depends on generating overall budgetary revenue and prioritizing the health budget to total government expenditure. Equation (1a) shows that public health expenditure (PHE_t_) is a proportion (*k*_*t*_) of total budgetary expenditure (*G*_*t*_) and shows the prioritization of health expenditure.

1 $${G}_{t}+{\gamma }_{t}{B}_{t-1}={T}_{t}+{B}_{t}+{A}_{t}+{O}_{t}$$

1a $${\mathrm{PHE}}_{t}={k}_{t}{G}_{t}$$

Literature on the development of fiscal space for health shows that prioritizing health expenditure to general government expenditure may not be a potential indicator of health financing because it can crowd out other uses of public spending [19]. Therefore, two other parameters, namely public health expenditure as a percentage of GDP and per capita public health expenditure, are suggested for use. These two can be considered a strong predictor of the health system’s dependence on out-of-pocket spending in resource-poor economies [18]. In addition, per capita public health expenditure and public health expenditure as a percentage of GDP also show the political commitment to health relative to other public spending [20].

Regarding the share of health expenditure to total government expenditure/GDP/population, Mathauer and Carrin [21] suggested a threshold level of spending to provide basic healthcare service packages. The threshold level of expenditure includes the following: the share of PHE should be 5% of GDP, the share of PHE should 15% of total government expenditure, and the share of PHE should be US$ 68 per capita of the total population. Mclntyre and Kutzin [20] argue that the achievement of PHE targets solely depends on the economy’s current fiscal capacity^1^ (revenue capacity and spending capacity), thereby minimizing the fiscal gaps and mobilizing more resources towards the health sector. However, a few have also argued that even if resource allocation is done at the different layers of the health system, a better understanding is needed of the relationship between organizational structures, systems and processes that influence evidence-based practices in the health system [22].

**Fig. 1:**
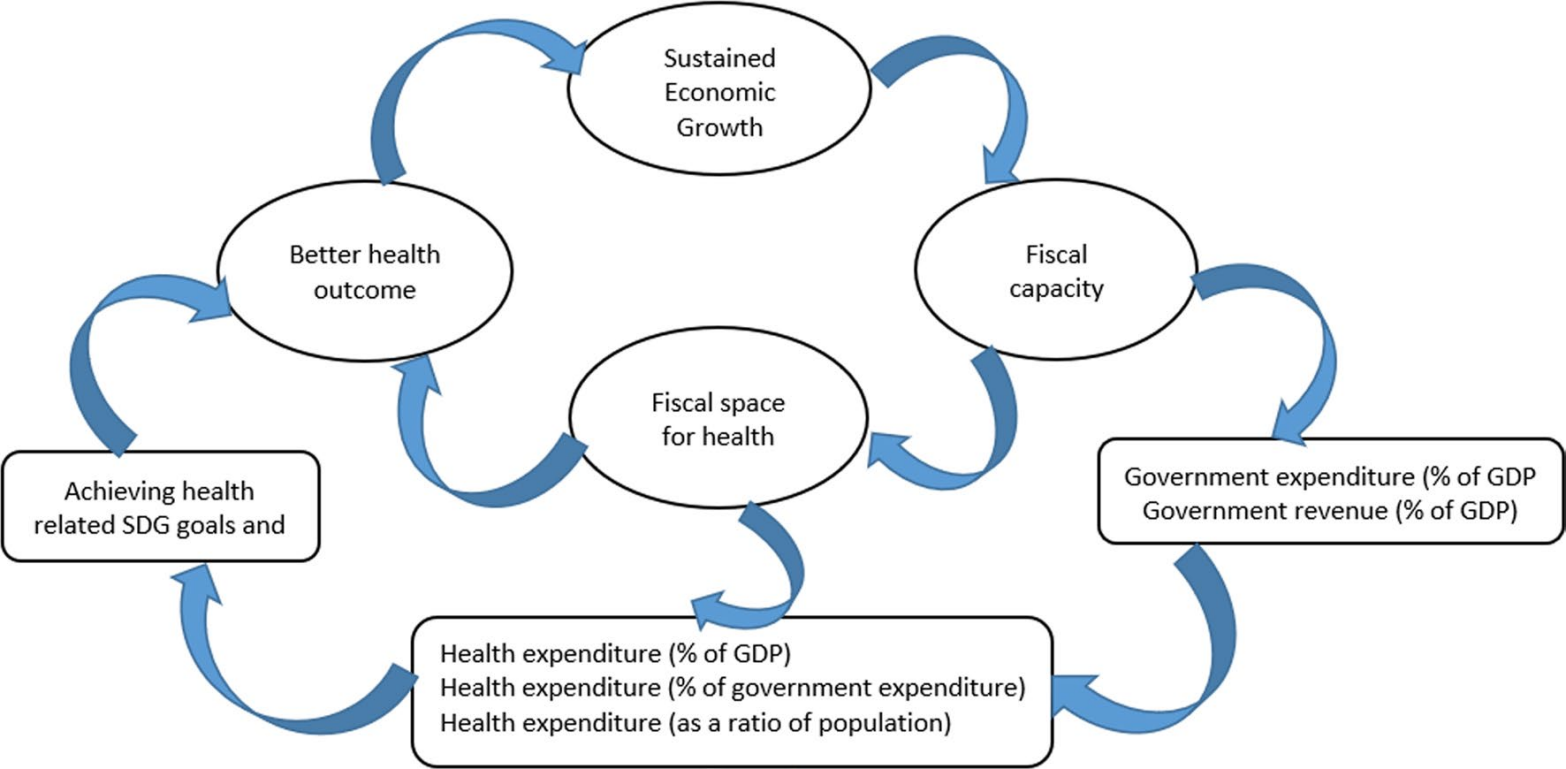
Linkages between economic growth, fiscal space and health outcomes. *Source* Authors’ representation

Figure 1 presents a conceptual framework of linkages between economic growth, fiscal space and health outcome based on the fiscal space literature [1, 4, 5, 20, 21]. Figure 1 also provides a possible roadmap for achieving better health outcomes and reducing the burden of out-of-pocket spending through conducive macroeconomic policies. It shows that favourable macroeconomic conditions such as increased economic growth and revenue generation lead to improved fiscal capacity of government [1, 4]. The fiscal capacity in turn leads to an increase in fiscal space for health financing, thereby reducing out-of-pocket spending and possibly improving health outcomes [20, 21]. This concept argues that these health financing strategies are interrelated [4, 5, 21]. For instance, unfavourable macroeconomic conditions during an economic crisis lead to lower revenue capacity and possibly reduce the fiscal space for health. Further, the reduced fiscal space for health could squeeze the developmental spending, thereby affecting health outcomes.

### Country-level experiences in the generation of fiscal space for health

Table 1 shows success stories of individual countries and groups of countries that have realized fiscal space for health through various fiscal policy measures and increased public expenditure on health.

**Table 1:** The linkage between fiscal policy and health

Country	Fiscal policy measures	Fiscal space for health channels
Uganda [21]	Increase domestic revenue and improve efficiency and absorptive capacity of grants	Domestic revenue mobilization
Nigeria [24]	Increase tax revenue from the oil sector and the utilization of existing resources	Alternative revenue mobilization
Bhutan [26]	Generate health-specific revenue (i.e. earmarked taxes)	Alternative revenue mobilization
Nepal [27]	Higher tax collection from payroll tax, value-added tax and excise duty	Alternative revenue mobilization
South-East Asia Region (SEAR) [28]	Increase tax revenues from earmarked and sin tax	Alternative revenue mobilization
Brazil, Russia, India, China and South Africa (BRICS) [32]	Smooth flow of central grants due to good coordination between central government and states	Prioritization of government
Bhutan [25]	Create an enabling environment for private health providers	Prioritization of government
Ghana [33]	Increase efficiency and absorptive capacity of health grants	Alternative revenue mobilization
Asian [37]	Higher excise duty on tobacco products (i.e. cigarettes and alcohol)	Alternative revenue mobilization
Indonesia [38]	Enhance the indirect tax base and increase nontax revenue by exploiting natural resources	Alternative revenue mobilization
South Africa [34]	Improving tax administration by minimizing tax evasion	Alternative revenue mobilization
Nepal [35]	Technological advancement in tax collection and minimizing leakage in health insurance payments to the poor by facilitating online bank transactions	Alternative revenue mobilization
Bangladesh [29]	Improve tax collection and prioritization of the health budget	Prioritization of government
Low- and middle-income countries (LMICs) [30]	Generate more nontax revenue through natural resources	Alternative revenue mobilization
Indian States [9]	Emphasize fiscal capacity by raising domestic revenue	Domestic revenue mobilization
India [6]	Compulsory tax-based financing system for the health sector	Prioritization of government
India [31]	Disease-specific and need-based financing grants from central government to states	Prioritization of government
India [39]	Strong political commitment to implementing health programmes for primary health services	Prioritization of government

*Source* Authors’ representation

The countries of Uganda, Nigeria, Bhutan, Nepal, Ghana, Bangladesh and Indonesia have increased their fiscal space for health through domestic revenue mobilization [23–27]. Similarly, the South-East Asia Region (SEAR) and many low- and middle-income countries (LMICs) have improved their health system financing through nontax revenue and external grants [28–30]. The SEAR and BRICS [Brazil, Russia, India, China and South Africa] countries have emphasized prioritizing health budgets through the smooth flow of external grants [30–32]. Ghana, South Africa and Nepal have increased fiscal space for health by improving efficiency and governance in tax structure and expenditure patterns [33–35]. The literature also argues that the medical system in emerging countries like China has disparities in health funding capacity between the economically less developed regions and economically developed regions, leading to poor healthcare programme implementation [36].

Table 1 suggests three crucial fiscal spaces for health channels: domestic revenue mobilization, alternative revenue mobilization and prioritization of health spending. A country can mobilize finance towards the health sector irrespective of economic development.

## Methods and descriptive analysis

This study follows a descriptive approach to analyse the trends and patterns of fiscal space for health performance. We use a linear trend (dummy variable regression) model in our empirical analysis. The dummy variable regression model compares the performance of fiscal space for health indicators across the four political regimes in India, namely NDA1 (1998–1999 to 2003–2004), UPA1 (2004–2005 to 2008–2009), UPA2 (2009–2010 to 2013–2014) and NDA2 (2014–2015 to 2020–2021). In a dummy variable regression model, the dummy coefficient will identify the differences if they exist between two time periods. However, they do not suggest the reasons for the differences [40]. The log-linear model for our empirical analysis is presented in Eq. (2).

2 $$\mathrm{Ln}{Y}_{t}={\beta }_{1}+{\beta }_{2}\mathrm{TREND}*\mathrm{UPA}1+{\beta }_{3}\mathrm{TREND}*\mathrm{UPA}2+{\beta }_{4}\mathrm{TREND}*\mathrm{NDA}2+{\mu }_{t},$$

where ${{\text{Ln}}{\text{Y}}}_{\text{t}}$= natural log of fiscal space indicators (see Table 5); $\mathrm{UPA}1$= 1 for the years from 2004–2005 to 2008–2009; otherwise UPA1 = 0; $\mathrm{UPA}2$= 1 for the years from 2009–2010 to 2013–2014; otherwise UPA2 = 0; $\mathrm{NDA}2$= 1 for the years from 2014–2015 to 2020–2021; otherwise NDA2 = 0.

The intercept ${\beta }_{1}$ captures the effect of the NDA1 regime (i.e. from 1998–1999 to 2003–2004) and the base category across political regimes and helps us avoid the dummy variable trap. Hence, the intercept ${\beta }_{1}$ represents the mean effect of particular fiscal space indicators during the NDA1 regime. The slope coefficients ${\beta }_{2}$, ${\beta }_{3}$ and ${\beta }_{4}$ in Eq. (2) are differential intercept coefficients because they present the mean of UPA1, UPA2 and NDA2 as compared to the base category NDA1.

This subsection analyses the health financing situation and a possible source of fiscal space in India. Earlier literature on health financing in India is sparse, and most evidence shows that public finance policies are an instrument for reducing poverty and promoting human development [41–43]. Studies have argued that fiscal consolidation measures such as higher state tax revenue mobilization, increased central tax share to states and reduced unproductive revenue expenditure would generate fiscal space for health [41]. Potential sources of revenue growth include increasing the tax base and reducing tax avoidance and evasion, which might increase the fiscal space among the Indian states [42]. In other words, increasing nontax (i.e. improving cost recovery) and tax revenues (i.e. if there is any scope) and reducing primary (noninterest) expenditure would generate fiscal space in the Indian economy [43].

As stated in the Constitution of India, health is a state subject. The maximum amount of funding is borne by the state government, and the central government usually supports family planning. Earlier literature argued that central government health grants through the National Health Mission (NHM) and higher revenue distribution of central tax share to states were major contributing factors of rising public expenditure across the Indian states [44]. Despite the surge in central revenue contribution to improve the states’ fiscal resources over the years, India continues to see stagnant growth in public expenditure on health [45].

**Fig. 2:**
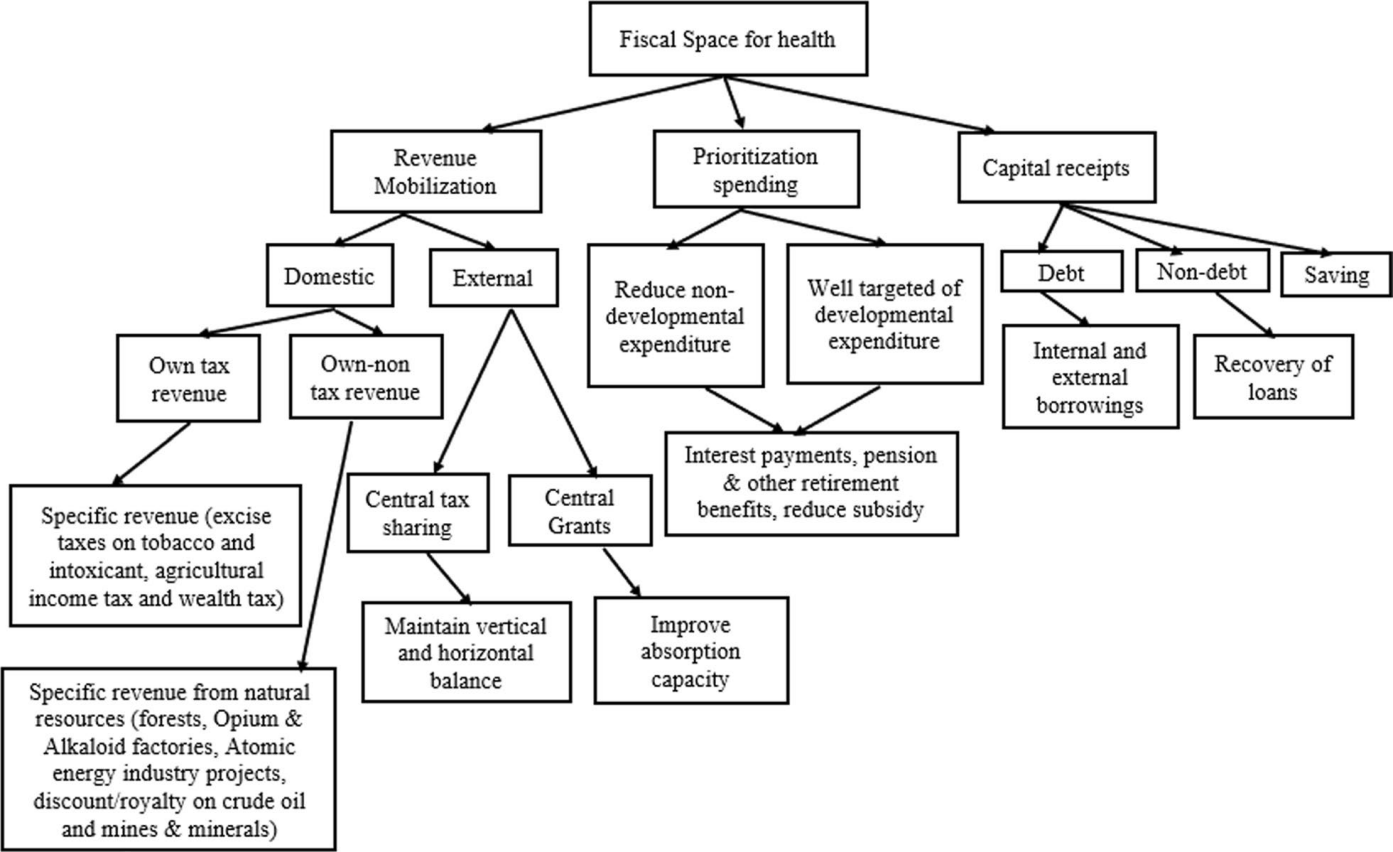
Possible fiscal space channels in the Indian context. *Source* Authors’ representation

Figure 2 presents a flow diagram of the possible fiscal space channels for health in the Indian context. Based on concepts in the literature, we have three major channels—revenue mobilization, reprioritization of expenditure, and borrowings—which are under the purview of the Ministry of Finance. First, revenue mobilization is divided into two parts: domestic sources and external sources. Then, domestic revenue mobilization is divided into two parts: the states’ tax revenue and the states’ nontax revenue. External sources of revenue are divided into two parts: central grants to states and central tax share to states. Figure 2 shows that state government could generate alternative revenue without depending on central grants, including tax revenue through increasing excise taxes on tobacco and intoxicants, imposing taxes on agricultural income and increasing taxes on wealth. These tax collections are feasible, and their share has been decreasing among the components of tax revenue in the state budget.

Similarly, specific nontax revenue generated by imposing more taxes on natural resources, opium and alkaloid factories, and atomic energy industry projects is important to reduce their adverse impact on the environment and health. Second, the prioritization of health spending in the budget is divided into reducing non-developmental expenditure and efficient utilization of developmental expenditure. Third, capital receipts are divided into borrowings and savings, an alternative financing mechanism to manage developmental expenditure during unfavourable macroeconomic conditions.

### Revenue mobilization channels

Table 2 presents sources of revenue mobilization flow in India from central and state governments for the period from 1998–1999 to 2020–2021. It shows that the central government generates revenue from the centre’s tax revenue and the centre’s nontax revenue. The share of tax revenue to total revenue was 70% in 1998–1999 and increased to 86.5% in 2020–2021. Similarly, the share of nontax revenue was 30% in 1998–1999 and decreased to 13.5% in 2020–2021. State government generates revenue from four sources: state tax revenue, state nontax revenue, central tax share to states and central grants to states. The share of state tax revenue to total revenue was 49.7% in 1998–1999 and decreased to 45% in 2020–2021; the share of the states’ nontax revenue to total revenue was 13.9% in 1998–1999 and decreased to 8.1% in 2020–2021; the states’ share from central taxes to total revenue was 22.8% in 1998–1999 and increased to 24.6% in 2020–21, and central grants to total revenue were 13.6% in 1998–1999 and increased to 22.3% in 2020–2021. Overall, India’s total revenue share to GDP was 8.4% in 1998–1999 and increased drastically to 36.1% in 2020–2021. The share of the centre’s revenue increased from 3.9 to 11.5% from 1998–1999 to 2020–2021, and the states’ revenue also increased from 4.5 to 24.6% over the period.

**Table 2:** Revenue mobilization channels in India (in %)

Year	Sources of revenue mobilization between central and state government								
	Central (as % total revenue)		State (as % total revenue)				Total revenue (as % GDP)		
	Tax	Nontax	Tax	Nontax	Central tax share	Central grants	Centre	State	India
1998–1999	70.0	30.0	49.7	13.9	22.8	13.6	3.9	4.5	8.4
1999–2000	70.7	29.3	48.9	14.5	21.7	14.9	4.4	4.9	9.2
2000–2001	71.0	29.0	48.8	13.3	21.8	16.0	4.5	5.4	9.8
2001–2002	66.3	33.7	49.4	12.6	20.9	17.1	4.4	5.5	9.9
2002–2003	68.7	31.3	50.0	12.8	20.7	16.5	4.9	5.8	10.7
2003–2004	70.9	29.1	49.8	12.0	21.7	16.4	5.2	6.1	11.3
2004–2005	73.5	26.5	50.1	12.8	21.6	15.5	5.6	6.6	12.2
2005–2006	77.9	22.1	49.3	11.1	21.8	17.8	5.9	7.3	13.2
2006–2007	80.8	19.2	47.6	11.9	22.7	17.8	6.8	8.3	15.1
2007–2008	81.1	18.9	45.9	12.4	24.3	17.4	7.9	9.1	16.9
2008–2009	82.1	17.9	46.3	11.8	23.2	18.7	7.6	9.8	17.4
2009–2010	79.7	20.3	47.3	11.6	21.5	19.7	7.5	10.0	17.5
2010–2011	72.3	27.7	49.3	9.8	23.5	17.5	9.5	11.3	20.8
2011–2012	83.8	16.2	50.7	9.0	23.3	17.0	8.6	12.6	21.2
2012–2013	84.4	15.6	52.3	9.4	23.3	15.1	9.5	13.6	23.1
2013–2014	80.4	19.6	52.0	9.7	23.2	15.0	10.4	14.0	24.3
2014–2015	82.0	18.0	49.0	9.0	21.2	20.8	10.5	15.1	25.6
2015–2016	79.0	21.0	46.2	8.4	27.6	17.8	10.5	16.1	26.6
2016–2017	80.1	19.9	44.6	8.3	29.7	17.4	11.2	16.6	27.8
2017–2018	86.6	13.4	48.7	7.7	26.1	17.5	10.9	17.7	28.6
2018–2019	84.8	15.2	46.4	8.3	28.5	16.8	11.1	18.7	29.8
2019–2020	80.6	19.4	45.6	8.1	23.9	22.4	11.6	20.2	31.7
2020–2021	86.5	13.5	45.0	8.1	24.6	22.3	11.5	24.6	36.1

*Source* Authors’ estimation from the Indian Public Finance Statistics and State Finance Report of the Ministry of Finance [13–15], Government of India. *Note*: GDP is calculated GDP at market prices at 2011–2012 base year prices

Overall trend analysis of revenue growth revealed two insights. First, central government resource mobilization to states increased substantially during the period. Second, the states’ revenue mobilization process is slow over the period, and around 47% of revenue is generated from central sources (i.e. tax share and grants). Therefore, states should emphasize generating domestic revenue through state tax and nontax revenue.

### Alternative revenue mobilization from domestic sources

Table 3 presents different sources of revenue that state governments can raise from the states’ own tax and nontax revenue. The states’ tax revenue source is agricultural income tax; taxes on professions, trades, callings and employment; taxes on property and capital transactions; and taxes on commodities and services. Table 3 shows that taxes on commodities and services are one of the major sources of state government revenue, which was 87.9% of the total state tax revenue in 2020–2021. Other sources of revenue are very sparse and should be improved. Revenue generation from nontax revenue could be an alternative channel for the state government that has no adverse impact on the income of the poor and is not regressive [46, 47]. Nontax revenue can be collected from interest payments, contributions from public sector units, economic services and social services. Table 3 shows that the source of revenue from interest payments decreased over the period, and there is enough space to generate revenue on these sources in India.

**Table 3:** Sources of revenue mobilization from domestic sources

Year	1998–1999	2003–2004	2004–2005	2009–2010	2013–2014	2014–2015	2015–2016	2020–2021
Sources of state tax revenue (% share)								
Agricultural income tax	0.3	0.0	0.0	0.0	0.0	0.0	0.0	0.0
Taxes on professions, trades, callings and employment	1.4	1.4	1.3	1.0	0.7	0.7	0.6	0.5
Taxes on property and capital transactions	9.8	11.5	11.9	12.4	12.3	12.1	12.4	11.6
Taxes on commodities and services	88.6	87.0	86.8	86.5	87.0	87.2	87.0	87.9
Sources of state nontax revenue (% share)								
Interest receipts	30.6	20.8	18.6	17.2	20.5	16.8	11.9	9.4
Dividends and profits	0.4	1.0	0.7	0.9	1.3	1.4	1.1	1.1
General services	22.5	25.1	23.8	27.1	14.7	17.3	21.2	25.5
Social services	7.3	8.8	7.6	10.2	17.3	18.0	20.1	16.5
Fiscal services	0.0	0.0	0.0	0.0	0.0	0.0	0.0	0.0
Economic services	39.1	44.3	49.3	44.7	46.1	46.4	45.8	47.5

*Source* same as Table 2

A few other alternative revenue sources include increased taxes on health hazard products and manufacturing companies (i.e. opium and alkaloid factories and atomic energy industry projects) and revenue from natural resources such as forests/rivers and mines/minerals that may improve the revenue capacity of the state government and increase fiscal space [42, 48]. Generating revenue from health hazard products, namely tobacco, could be an alternative revenue source, but it has some administrative and political complications to rationalize the tax structure [49]. Further, health policy decisions in India are made by multiple layers in state government, and always depend on the fiscal resource transfer policies of the central government. The literature argues that through health technology assessment (HTA), government can make evidence-based policy in terms of resource prioritization and utilization of funds at the local level [50].

### Expenditure prioritization channels

Table 4 shows the individual expenditure share to total revenue expenditure of Indian states. It can be seen that the percentage of medical and public health expenditure to total expenditure was 4.8% in 2020–2021. The trends show stagnant growth in public health expenditure over the period. Similar stagnant growth was seen in family welfare, water supply and sanitation expenditure. Figure 2 shows the possible sources of expenditure prioritization in India. We found that the government can prioritize the health sector by reducing the share of non-developmental to total spending. Some non-developmental expenditures can be reduced, including interest payments, pensions and other retirement benefits, and social security and welfare schemes.

**Table 4:** Expenditure reprioritization (% of revenue expenditure)

Year	Medical and public health	Family welfare	Water supply and sanitation
1998–1999	4.8	0.9	2.4
2003–2004	3.8	0.7	1.7
2004–2005	3.8	0.6	1.7
2008–2009	3.8	0.7	1.5
2013–2014	4.1	0.7	1.1
2014–2015	4.3	0.9	1.3
2015–2016	4.4	0.9	1.5
2020–2021	4.8	0.9	1.3

*Source* same as Table 2

Additionally, a few developmental expenditures such as subsidies can be reduced by curbing leakage. Because the share has been increased over the years, it might affect the prioritization of health spending. Earlier studies argued that reducing non-meritorious expenditure and increasing the efficient utilization of existing resources are possible ways to increase fiscal space [17, 30]. Further, a few studies have discussed the expenditure management channels to reduce the effective utilization of health funding and reduce the unwanted medical cost to the public [51]. Hassan et al. [36, 51] argued in the context of China that healthcare costs could be reduced by encouraging generic medicine use and manufacturing, improving the distribution of hi-tech medical equipment, and strict regulation of insurance payments. Therefore, the government should focus on the optimum utilization of health expenditure and consistently mobilize more funds towards health sector development.

### Borrowing channels

**Fig. 3:**
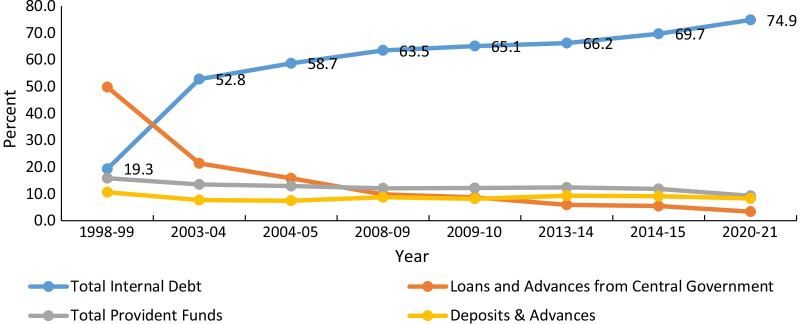
Sources of borrowing (as % total borrowings)

Figure 3 shows various sources of capital receipts—borrowings, savings and loans—that could be a potential revenue channel to meet the developmental expenditure during budget deficits (i.e. expenditure exceeds revenue). Earlier studies argue that borrowings are not viable for financing the deficit because they are always tied to debt service costs, thereby reducing the developmental expenditure [52]. Some studies argue that borrowings and savings are usually recognized as sources of revenue for fiscal space for health during the period of fiscal imbalance in LMICs [46, 47].

The most recent data show that savings and non-debt capital receipts have been reduced at the central level since 1990–1991, which should be increased because savings would generate investment and economic growth [9, 45]. At the state level, internal debt (market borrowings) is the major share of capital receipts, which increased from 19.3% in 1998–1999 to 74.9% in 2020–2021, and this excessive borrowing may have adverse effects on the prioritization of health expenditure.

## Empirical results

Table 5 presents the political regime-wise trend analysis of fiscal space indicators in India from 1998–1999 to 2020–2021. We have divided indicators into three components—revenue mobilization, expenditure prioritization and borrowings—across four time periods. We observe three important findings. First, central grants and shares in central taxes have increased over the period, and a significant increase is seen during the NDA2 regime. Second, the percentage of nontax revenue is stagnant over the period and should be increased. Third, health and overall developmental expenditures have increased substantially since 2009 in both the UPA2 and NDA2 regimes. Fourth, both gross fiscal deficit and domestic debt increased more during both UPA2 and NDA2 than in the previous period. The last 5 years of UPA2 (2009–2013) had confronted economic crises such as mounting current account deficits, high fiscal deficits and persistently high inflation [53].

**Table 5:** Trends of fiscal space indicators across political regimes (as a % of GDP)

Fiscal space indicators	NDA1 1998–2003	UPA1 2004–2008	UPA2 2009–2013	NDA2 2014–2020
Revenue mobilization				
Central tax revenue	3.2	5.4	7.3	9.1
Central nontax revenue	1.4	1.4	1.8	1.9
State tax revenue	2.7	3.9	6.2	8.5
State nontax revenue	0.7	1.0	1.2	1.5
Grants from the centre	0.8	1.4	2.0	3.6
Share in central tax	1.2	1.9	2.8	4.8
Expenditure prioritization				
Developmental expenditure	7.2	9.9	16.0	21.5
Non-developmental expenditure	6.6	8.1	11.2	14.7
Health expenditure	0.5	0.5	0.7	1.3
Subsidies	0.7	1.1	2.4	2.4
Defence expenditure	1.1	1.4	1.9	2.1
Administrative services	0.6	0.6	1.0	1.3
Pension and misc. general services	0.7	0.9	1.5	2.2
Interest payments	2.3	2.4	3.2	4.2
Borrowings				
Gross fiscal deficit	4.6	4.2	7.5	8.6
External liabilities of the centre	4.3	3.3	3.5	3.7
Domestic liabilities of centre and states	34.9	48.1	63.9	90.6

*NDA* National Democratic Alliance; *UPA* United Progressive Alliance government

Additionally, no economic reforms or innovative fiscal measures were initiated during the UPA2 regime from the perspective of sustained macroeconomic stability and revenue mobilization. This policy paralysis in the Indian economy was created through unwanted subsidies and inefficient expenditure management during the UPA2 regime [15]. Similar trends were exhibited in the NDA2 regime regarding lower economic growth, but many reforms have been taken in terms of taxation, spending and fiscal policy [15]. However, the unprecedented COVID-19 epidemic crisis has resulted in poor economic growth, high deficit and high domestic debt during the NDA2 period. On the contrary, expenditure prioritization has increased during the COVID-19 period in India [15].

### Trend of growth in fiscal space indicators across political regimes in India

Table 6 presents the growth trend in fiscal space indicators across political regimes using a dummy variable regression model. The regression result shows that the mean impact of the intercept (NDA1) on health expenditure is about 9.921%, implying that the impact on public health expenditure is higher by 0.442%, 1.140% and 2.084% in UPA1, UPA2 and NDA2, respectively. In other words, the mean impact of public health expenditure was higher during the UPA2 regime (2009–2013) and NDA2 regime (2014–2020). This implies that health expenditure increased over the period irrespective of political regime, but growth is double in the NDA2 regime. The mean impact of intercept on central government transfer to states (i.e. grants and tax share) is around 22%, while the effects in the NDA2 and UPA2 regimes were higher. This implies that the mobilization of revenue towards states increased during the NDA2 regime compared to the UPA2 and UPA1 regimes.

**Table 6:** Log-linear trend regression of fiscal space indicators (independent variable: time)

Dependent variables	Mean of the base category	Differential mean parameters			
	${\beta }_{1}$ = NDA1	${\beta }_{2}$ = UPA1	${\beta }_{3}$ = UPA2	${\beta }_{3}$ = NDA2	*R*-squared
Real GDP	15.30*** (−0.043)	0.358*** (−0.064)	0.678*** (−0.064)	1.055*** (−0.058)	0.948
Central tax revenue	11.85*** (−0.089)	0.870*** (−0.133)	1.510*** (−0.133)	2.107*** (−0.122)	0.944
Central nontax revenue	11*** (−0.14)	0.2 (−0.207	1*** (−0.207)	1.286*** (−0.191)	0.758
State tax revenue	11.67*** (−0.098)	0.747*** (−0.145)	1.522*** (−0.145)	2.222*** (−0.134)	0.941
State nontax revenue	10.34*** (−0.085)	0.686*** (−0.127)	1.216*** (−0.127)	1.819*** (−0.117)	0.932
Grants from the centre	10.52*** (−0.116)	0.883*** (−0.173)	1.570*** (−0.173)	2.481*** (−0.159)	0.932
Share in central tax	10.84*** (−0.109)	0.829*** (−0.161	1.565*** (−0.161)	2.461*** (−0.148)	0.94
Developmental expenditure	12.67*** (−0.096)	0.666*** (−0.142)	1.478*** (−0.142)	2.144*** (−0.131)	0.94
Non-developmental expenditure	12.58*** (−0.082)	0.566*** (−0.123)	1.206*** (−0.123)	1.856*** (−0.113)	0.94
Public health expenditure	9.921*** (−0.086)	0.442*** (−0.128)	1.140*** (−0.128)	2.084*** (−0.118)	0.949
Subsidies	10.35*** (−0.14)	0.730*** (−0.208)	1.874*** (−0.208)	2.194*** (−0.191)	0.894
Defence expenditure	10.83*** (−0.063)	0.559*** (−0.094)	1.205*** (−0.094)	1.680*** (−0.0863)	0.957
Administrative services	10.12*** (−0.087)	0.460*** (−0.13)	1.230*** (−0.13)	1.910*** (−0.119)	0.939
Pension services	10.28*** (−0.106)	0.633*** (−0.157)	1.476*** (−0.157)	2.261*** (−0.144)	0.936
Interest payments	11.53*** (−0.078)	0.408*** (−0.116)	0.999*** (−0.116)	1.645*** (−0.106)	0.934
Gross fiscal deficit	12.23*** (−0.088)	0.235* (−0.131)	1.154*** (−0.131)	1.677*** (−0.12)	0.928
External debt	12.15*** (−0.058)	0.108 (−0.086)	0.490*** (−0.086)	0.915*** (−0.079)	0.894
Domestic debt	14.24*** (−0.095)	0.690*** (−0.142)	1.292*** (−0.142)	2.012*** (−0.131)	0.931

All variables are real constant prices at base 2011–2012. Standard errors are in parentheses

*Source* Authors’ estimation

* and *** denote the significance level at 10% and 1%, respectively. The actual mean impact of UPA1, UPA2 and NDA2 can be obtained by adding a differential mean of estimated coefficients (${\beta }_{2}$, ${\beta }_{3}{\beta }_{4}$) with the mean coefficient (${\beta }_{1}$) of the base category NDA1

By comparing the differential means of UPA1, UPA2 and NDA2 with the mean of the base category NDA1, this study has revealed the following insights. First, economic growth, health expenditure, central tax share and central grants increased during the four political regimes, but the differences are less than the base category. Second, non-debt receipts in terms of recovery of loans show an increasing trend over the period but are statistically significant during the NDA2 regime. Third, debt receipts in terms of both domestic and external borrowings show an increasing trend over the period and statistical significance during the NDA2 regime. Fifth, some non-developmental expenditures—administrative expenditure, pensions, interest payments—have increased over the period, but the growth is greater in particular in the NDA2 regime. Sixth, some developmental expenditures—subsidies and defence expenditure—have increased over the period, and the increase is greater during the NDA2 than the UPA regimes.

Our finding is the corollary to the international evidence that argues that increasing tax and nontax revenue, increasing central grants and prioritization of health expenditure are possible sources of fiscal space for health [23, 24, 28, 39]. On the contrary, we have found that interest payments, defence expenditure and debt have become a larger share of total revenue expenditure over the period. Both the UPA2 and NDA2 regimes have tried to improve revenue mobilization by discharging more finance to states. This trend can easily be seen during the NDA2 regime, where expenditure prioritization on health has increased despite lower economic growth and high fiscal deficit. This could be because of the COVID-19 crisis that led to increased health budget allocation, as the Indian government has announced several reforms to tackle the COVID-19 crisis by strengthening the health system in India [13]. Literature in the Indian context has pointed out many drawbacks in the Indian health system from a governance perspective [54]. Studies have suggested that an integrated national health data system, improved purchasing and regulation in the private sector, and intersectoral delivery of health services are required to improve the health system across the level of government.

## Discussion and conclusions

### Fiscal space commitment and health policy across political regimes in India

Table 7 shows the political commitment of the present government by analysing the fiscal policy intervention on the generation of fiscal space for health. To analyse the political commitment, we have purposefully gathered information related to health sector development and fiscal policies from the annual budget report and speeches of the finance ministry during the annual budget presentation. We found that the central government has initiated various fiscal policy interventions in revenue mobilization, expenditure prioritization, and efficiency and governance to generate fiscal space in the economy. Some bold steps have been taken by the NDA2 government which could be an alternative strategy for fiscal space, such as one-nation one-tax policy as goods and services tax (GST) reform for increasing the tax base among states, reducing untargeted subsidies on natural gas, spending priority on the social sector, tax administration reform, and reducing corruption and black money through demonetization. Similarly, the central government has prioritized the health sector by introducing various health-related programmes such as Swachh Bharat,^2^ Ayushman Bharat,^3^ Jan Aushadhi and Jan Suraksha health insurance schemes. Additionally, health-related areas such as infrastructure, development, health outcomes and national health insurance^4^ for financial protection have also been priority areas in the last 7 years of the NDA2 government.

**Table 7:** Fiscal policy intervention and health programme initiatives across political regimes

Budget year	Finance minister	Ruling party	Fiscal space commitment	Health prioritization
1998–1999	Mr Yashwant Sinha	NDA	Decentralization and expenditure restructuring	Plan allocation for the Ministry of Health and Family Welfare (MoHFW) increased but was not prioritized
1999–2000	Mr Yashwant Sinha	NDA	Expenditure management	The National Human Development Initiative (NHDI)
2000–2001	Mr Yashwant Sinha	NDA	Introduced a single-rate central value-added tax (CENVAT), and medical items were exempted	National population policy and recognizing Indian systems of medicine
2001–2002	Mr Yashwant Sinha	NDA	Fiscal consolidation measures to reduce the fiscal deficit	Recognized the need for investment in the social sector
2002–03	Mr Yashwant Sinha	NDA	Introduced Fiscal Responsibility and Budget Management Act (FRBM) bill for fiscal consolidation	Increase allocation in the Indian system of medicine
2003–2004	Mr Jaswant Singh	NDA	Fiscal consolidation and debt management	Introduced the community-based Universal Health Insurance Scheme
2004–2005	Mr P. Chidambaram	UPA	Special economic packages were announced for poor states to improve their fiscal position	Group health insurance schemes under the national common minimum programme
2005–2006	Mr P. Chidambaram	UPA	Implemented 12th Finance Commission recommendations on tax sharing and grants to states	Launched the National Rural Health Mission (NRHM) programme
2006–2007	Mr P. Chidambaram	UPA	Modernization of tax administration	Strengthening the NRHM at the block level
2007–2008	Mr P. Chidambaram	UPA	Introduced national-level goods and services tax (GST)	11th 5-year target to increase health expenditure to 2–3% of GDP
2008–2009	Mr P. Chidambaram	UPA	Sustained growth rate and high fiscal revenue collection	Introduced the Rashtriya Swasthya Bima Yojana (RSBY) insurance scheme and the National Programme for Health Care of the Elderly
2009–2010	Mr Pranab Mukherjee	UPA	Increase tax effort through administrative measures	Introduced the Aam Aadmi Bima Yojana (AABY)
2010–2011	Mr Pranab Mukherjee	UPA	Consolidating growth and tax reforms through GST	Prepared district health profiles of all districts through the Inclusive Development programme
2011–2012	Mr Pranab Mukherjee	UPA	High growth and many social welfare schemes introduced	RSBY scheme extended to Mahatma Gandhi National Rural Employment Guarantee Act (MGNREGA) beneficiaries
2012–2013	Mr Pranab Mukherjee	UPA	Implement the Medium-term Expenditure Framework	Launched Pradhan Mantri Swasthya Suraksha Yojana (PMSSY)
2013–2014	Mr P. Chidambaram	UPA	Emphasis on creating fiscal space by reducing the fiscal deficit, current account deficit	National Health Mission introduced by adding National Urban Health Mission
2014–2015	Mr Arun Jaitley	NDA	Minimum government and maximum governance approach for expenditure management	Health for All initiatives; Swachh Bharat Abhiyan
2015–2016	Mr Arun Jaitley	NDA	Good governance initiatives	Improving the quality of life and public health through Swachh Bharat initiatives
2016–2017	Mr Arun Jaitley	NDA	Governance and fiscal discipline; simplification and rationalization of taxation	Niramaya health insurance scheme and PMJAY health insurance scheme launched
2017–2018	Mr Arun Jaitley	NDA	Measures for stimulating growth; transparency in electoral funding and GST implementation	Sabka Saath Sabka Vikas empower rural women with health and nutrition, employment
2018–2019	Mr Arun Jaitley	NDA	Introduced health and education cess	National Health Protection Scheme
2019–2020	Mrs Nirmala Sitharaman	NDA	Measures to widen and deepen the tax base	Health society through Ayushman Bharat initiatives
2020–2021	Mrs Nirmala Sitharaman	NDA	Digital governance	A holistic vision of healthcare for wellness, water and sanitation
2021–2022	Mrs Nirmala Sitharaman	NDA	Atma Nirbhar packages for structural reform	Pradhan Mantri Garib Kalyan Yojana (PMJAY) during lockdown due to COVID-19

*Source* Authors’ estimation from budget documents, Ministry of Finance [13], Government of India

### NDA1 regime (1998–1999 to 2003–2004)

#### Decentralization and expenditure restructuring

The central government appointed a special task force to examine and recommend measures for devolution of additional financial powers to the states and additional or alternative means by which states could raise more resources. Additionally, it appointed a task force to examine the distinction between plan and non-plan expenditure and better function of the central sector and centrally sponsored schemes.

#### Expenditure management

Expenditure management constitutes an expenditure reforms commission to reduce unwanted expenditure, especially non-developmental expenditure; thereby, the government has reduced the role and administrative structure of the government.

#### Fiscal consolidation and debt management measures

High fiscal deficit was a severe issue in 2002–2003, around 10% of GDP. During that time, the government borrowed INR 111,000 crore to meet the financial commitment. Therefore, the government introduced a fiscal responsibility bill in parliament. Fiscal consolidation was done through revenue enhancement under modern tax administration and expenditure rationalization. For debt management, they introduced debt swap schemes and provided nonperforming asset (NPA) schemes.

#### National Human Development Initiative (NHDI)

The central government provides access to five basic requirements of life, namely food, healthcare, education, employment and shelter. In healthcare in particular, they have created more primary healthcare centres in most rural areas and integrated all central ministry schemes related to health and family welfare for better access.

#### National population policy and recognizing Indian systems of medicine

The objective of the national health policy was to reduce the total fertility rates to replacement level by 2010. The government recognized the role of the Indian systems of medicine and homeopathy in our healthcare.

#### Community-based Universal Health Insurance Scheme (UHIS)

Th objective of this scheme was to provide easy access to good health services to families living below the poverty line (BPL). This scheme provided for the following reimbursements: a premium equivalent to Re 1 per day (or Rs 365 per year) for an individual, Rs 1.50 per day for a family of five, and Rs 2 per day for a family of seven for medical expenses up to Rs 30,000 towards hospitalization, coverage for accidental death of Rs 25,000, and compensation due to loss of earnings at a rate of Rs 50 per day up to a maximum of 15 days.

### UPA1 and UPA2 regimes (from 2004–2005 to 2013–2014)

#### Group health insurance schemes

Access to medical care is not easily available to the poor, and UHIS was skewed favouring the nonpoor. The government introduced a new group health insurance scheme (GHIS). Under the GHIS, the premium will be Rs 120 per person, but the insurance coverage would be for a total of Rs 10,000.

#### National Rural Health Mission (NRHM)

The focus of the NRHM was to strengthen primary healthcare through grassroots-level public health interventions based on community ownership.

#### Rashtriya Swasthya Bima Yojana (RSBY)

RSBY health insurance provided health coverage of INR 30,000 for every worker in the unorganized sector, falling under the BPL family category.

#### National Programme for Health Care of the Elderly

Under this programme, two national institutes of ageing, eight regional centres, and a department for geriatric medical care in one medical college hospital in each state were implemented.

#### Aam Aadmi Bima Yojana (AABY)

The AABY scheme was introduced for death and disability coverage of rural landless in the country in conjunction with the state government, and the scheme covered 6.032 million lives.

#### Pradhan Mantri Swasthya Suraksha Yojana (PMSSY)

The PMSSY aimed to set up All India Institute of Medical Sciences (AIIMS)-like institutions. The upgrading of existing government medical colleges was expanded to cover the upgrade of seven more government medical colleges. Thus, the PMSSY has enhanced the availability of affordable tertiary healthcare.

### NDA2 regime (2014–2015 to 2020–2021)

#### Health for All initiatives and Swatch Bharat Abhiyan

The government intended to provide every household with total sanitation by the year 2019, the 150th anniversary of the birth of Mahatma Gandhi, through the Swachh Bharat Abhiyan. To move towards “Health for All”, two key initiatives were launched, namely the Free Drug Service and Free Diagnostic Service, to be taken up on priority.

#### ***Good governance initiatives through HTA***^5^

Many good governance measures were introduced to reduce leakage in subsidies and inefficiency in delivering many welfare schemes and introduced a direct transfer mechanism to minimize leakage in subsidies and to target needy people. Additionally, the government of India recently committed to institutionalizing HTA as an integral component of the health resource allocation decision-making process [55, 56]. Similarly, HTA shows a more significant impact on evidence-based policy-making and prioritization of the health system to the needs of the local level [55, 56].

#### Pradhan Mantri Jan Arogya Yojana (PMJAY)

The National Health Protection Scheme covers over 100 million poor and vulnerable families (approximately 500 million beneficiaries), providing coverage up to 5 lakh rupees per family per year for secondary and tertiary care hospitalization.

#### Pradhan Mantri Garib Kalyan Yojana

During lockdown due to COVID-19, the Prime Minister announced the Pradhan Mantri Garib Kalyan Yojana, valued at 2.76 lakh crores. This provided free food grain to 800 million people, free cooking gas for 80 million families for months, and cash directly to over 400 million farmers, women, elderly, the poor and the needy.

#### Atma Nirbhar packages announced

The government announced Atma Nirbhar package structural reforms. Additionally, a few reforms have taken place during the COVID-19 era, such as the redefinition of micro, small and medium enterprises (MSMEs), commercialization of the mineral sector, agriculture and labour reforms, and privatization of public sector undertakings. One Nation One Ration Card and production-linked incentive schemes were notable reforms carried out during this period. Faceless income tax assessment, direct benefit transfer (DBT) and financial inclusion were also fiscal reforms announced under Atma Nirbhar packages.

#### Fiscal space-related commitment

The government initiated a few revenue mobilization reforms that include stable taxation policy and non-adversarial tax administration; proposed health-specific tax^6^; imposed earmark tax^7^; expenditure prioritization^8^; and utilization of central transfer.^9^

This study is an initial attempt to identify possible sources of fiscal space for health in India and examine the fiscal commitment towards health sector development by estimating the trend growth of fiscal space indicators over the political regime from 1998–1999 to 2020–2021.

By synthesizing the evidence from the literature, we identified four important fiscal space channels (i.e. domestic revenue mobilization, health-specific revenue mobilization, prioritization of health and good governance mechanisms) through which India could generate fiscal space for the health sector. The linear growth of fiscal space indicators over the political regimes showed that increasing tax and nontax revenue, reducing subsidies and interest payments, and improving central grants are possible sources of fiscal space for health in India.

It was seen that the NDA2 regime has made greater effort to improve fiscal space in the economy than the previous two political regimes, NDA1 and UPA, in terms of revenue mobilization, expenditure prioritization, improved efficiency and good governance in expenditure management. Some bold steps have been taken by the NDA2 regime which could be an alternative strategy for fiscal space, including one-nation one-tax policy on indirect tax reform, imposing a health-specific tax (i.e. health CESS), higher excise duty on tobacco products, prioritizing subsidies on cooking gas connection to poor people, tax administration reform and direct financial transfer to beneficiaries.

Based on the evidence generated from the analysis of parliament speeches on fiscal space for health commitment, we found that the central government has the political will to prioritize the health sector. Some health-related commitments were announced, such as the Swachh Bharat programme of hygiene and cleanliness, and Ayushman Bharat, a national health protection scheme for faster progress towards universal health coverage.

We can conclude that the central government has a political commitment to generating revenue through various fiscal policy reforms, and over the period, health has been prioritized. Still, there is less evidence on health-related political commitment for an increased share of health expenditure to total budgetary allocation. But in the last 2 years, the health budget has been prioritized due to the COVID-19 pandemic crisis despite slower economic growth in India.

Like other studies, this study is not free from limitations, which include an experimental approach to evaluate the fiscal commitment to health and prioritization from an implementation perspective. Therefore, evaluating the impact of fiscal policy interventions on health prioritization and health outcomes is not within the scope of our analysis and could be a helpful direction for further research on fiscal space. Despite its limitations, this study can serve as a policy document for fiscal space analysis from the political-economic perspective. The role of the Ministry of Finance can be assessed through budget documents.

## Data Availability

Data are available in the public domain for research purposes, not for commercial use. Data can be obtained from the available government documents and budget reports from the Ministry of Finance of the Government of India.

## Abbreviations

COVID: Coronavirus disease
ICT: Information and communications technology
GDP: Gross domestic product
FRBM: Fiscal Responsibility and Budget Management Act
NDA: National Democratic Alliance
UPA: United Progressive Alliance
PHE: Public health expenditure
LMICs: Low- and middle-income countries
SEAR: South-East Asia Region
BRICS: Brazil, Russia, India, China and South Africa
NHM: National Health Mission
HTA: Health technology assessment
GST: Goods and service tax
NPA: Nonperforming assets
NHDI: National Human Development Initiative
UHIS: Universal Health Insurance Scheme
NRHM: National Rural Health Mission
RSBY: Rashtriya Swasthya Bima Yojana
AABY: Aam Aadmi Bima Yojana
PMSSY: Pradhan Mantri Swasthya Suraksha Yojana
PMJAY: Pradhan Mantri Jan Arogya Yojana
MSMEs: Micro, small and medium enterprises
MGNREGA: Mahatma Gandhi National Rural Employment Guarantee Act

## Footnotes

1 The rules of thumb for fiscal capacity (as % GDP) are as follows: very low level of fiscal capacity (< 15%), low fiscal capacity (15–20%), low to medium (20–25%), medium (25–35%), medium to high (35–45%) and very high (> 45%). The current revenue capacity (as % GDP) and spending capacity (as % GDP) indicate the current scope for increasing fiscal space for health [20].

2 Swachh Bharat is not only a programme of hygiene and cleanliness but, at a deeper level, a programme for preventive healthcare and building awareness. Under the Swachh Bharat scheme, the government has already constructed more than 60 million toilets. The government had planned to construct around 20 million toilet facilities by 2020 [13].

3 Under the Ayushman Bharat PMJAY programme, the government provides healthcare services in primary, secondary and tertiary care hospitals [13]. Primary healthcare is provided through health and wellness centres. This ensures enhanced productivity and well-being and averts wage loss and impoverishment. These schemes will also generate hundreds of thousands of jobs, particularly for women. The government is steadily but surely progressing towards the goal of universal health coverage.

4 The national health insurance scheme covers over 100 million poor and vulnerable families (approximately 500 million beneficiaries), providing coverage up to 0.5 million per family per year for secondary and tertiary care hospitalization [13].

5 HTA provides a globally accepted approach to synthesizing evidence-based policy on cost and clinical effectiveness through an equity and ethics lens [55].

6 The Finance Minister in his budget speech stated, “While undertaking all these measures (health programmes), I also need to mobilize resources. Accordingly, I propose to increase the specific excise duty on cigarettes in the range of 11–72%. Similar increases are proposed on cigars, cheroots, and cigarillos. Likewise, the excise duty is being increased from 12 to 16% on pan masala, from 50 to 55% on unmanufactured tobacco and from 60 to 70% on gutkha and chewing tobacco. I also propose to levy an additional duty of excise at 5% on aerated waters containing added sugar. Further, the imposition of clean energy cess levied on coal, lignite, and peat under Swachh Bharat schemes. These are healthy measures and I hope everyone would welcome from a human health point of view.”

7 The Finance Minister in his budget speech stated, “Madam Speaker, at present there is a 3% CESS on personal income tax and corporation tax consisting of 2% CESS for primary education and 1% CESS for secondary and higher education. To take care of the needs of education and health of BPL and rural families, I propose to increase the CESS by 1%. The existing 3% education CESS will be replaced by a 4% ‘Health and Education CESS’ to be levied on the tax payable. This will enable us to collect an estimated additional amount of 110000 Million.”

8 “Despite the consequential reduced fiscal space for the centre, the government has decided to continue supporting important national priorities such as agriculture, education, health, MGNREGA (Mahatma Gandhi National Rural Employment Guarantee Act) and rural infrastructure, including roads. Programmes targeted for the poor and the underprivileged will be continued by us.”

9 “Large increase in central tax devolution to states would improve the fiscal space for state government. I urge states to utilize their enhanced resources effectively in these areas (national priority especially the health sector). I assume that the state government will be spent on these areas by using the fiscal space and improve the share of spending on these programmes.”
